# Exploring Radiographers’ Engagement in Research: Motivation and Barriers in Five Arab Countries

**DOI:** 10.3390/healthcare11202735

**Published:** 2023-10-13

**Authors:** Mohamed M. Abuzaid, Nissren Tamam, Wiam Elshami, Manal Ibham, Mohammed Aljamal, Simaa Khayal, Ahmed Abdullah, Zuhal Y. Hamd, Malene Roland Vils Pedersen

**Affiliations:** 1 Medical Diagnostic Imaging Department, College of Health Sciences, University of Sharjah, Sharjah 27272, United Arab Emirates; 2 Research Institute for Medical and Health Sciences, University of Sharjah, Sharjah 27272, United Arab Emirates; 3 Department of Physics, College of Sciences, Princess Nourah bint Abdulrahman University, Riyadh 11564, Saudi Arabia; 4 Department of Medical Imaging, Faculty of Allied Medical Sciences, Arab American University, Jenin 11184, Palestine; 5 Independent Researcher, D04 T6F4 Dublin, Ireland; 6 College of Medicine, University of Diyala, Diyala 32008, Iraq; 7 Department of Radiological Sciences, College of Health and Rehabilitation Sciences, Princess Nourah bint Abdulrahman University, Riyadh 11671, Saudi Arabia; 8 Department of Radiology, University Hospital of Southern Denmark, 7100 Vejle, Denmark; 9 Department of Regional Health Research, University of Southern Denmark, 5230 Odense, Denmark

**Keywords:** radiographers, research, Arab countries, research engagement, professional development, motivation

## Abstract

(1) Background: This study aims to comprehensively understand the motivations driving radiographers in five Arab countries to engage in research. (2) Methods: A cross-sectional study employing an anonymous online survey was conducted for 12 weeks from May to July 2023. The study sample consisted of 250 radiographers, with equal representation from Iraq, the Kingdom of Saudi Arabia, Palestine, Sudan, and the United Arab Emirates. (3) Results: Overall, the participants showed limited involvement in research-related activities in all five countries, particularly in presenting at conferences and publishing in peer-reviewed journals. Most participants believed research positively impacts their professional development (34.8%) and patient care and outcomes (40%). The participants perceived professional development (36.4%) as a key motivator for research engagement. A significant majority (81.6%) expressed motivation to start research in clinical practice. A total of 66.8% found research opportunities available during clinical practice. Barriers included time constraints (56%), limited resources (47.2%), and lack of support and skills (33.2% and 32%, respectively). (4) Conclusion: This study emphasises the need for targeted strategies to enhance research engagement among radiographers in the Arab region. Addressing barriers, such as time constraints and resource limitations, while leveraging intrinsic motivators, such as professional development, is crucial for fostering a culture of research-driven excellence in radiography.

## 1. Introduction

In the evolving healthcare industry, radiographers, who are at the crossroads of medical imaging and patient care, have great potential to lead research, refine imaging techniques, and contribute to healthcare advancement through their unique expertise in acquiring and interpreting diagnostic images. Radiographers have diverse research opportunities, such as refining imaging protocols, enhancing image quality, optimising radiation doses, improving education methods, advancing image interpretation techniques, integrating AI into medical imaging, and contributing to developing cutting-edge imaging technologies. Their research bridges theoretical knowledge and practical applications, harnessing their unique insights into patient interactions, clinical workflows, and imaging techniques. These efforts lead to substantial advancements that directly benefit patient outcomes and contribute significantly to the field [1,2,3,4,5,6,7,8,9,10].

Radiographers’ motivations for research can be intrinsic or extrinsic. Intrinsic motivations include personal development, new challenges, satisfaction, and funding. Extrinsic motivations include recognising research’s potential to empower and elevate the profession. However, barriers such as limited time, funding, and leadership support hinder their involvement. Strategies to improve access to postgraduate education and research support are recommended [11,12,13,14,15].

Despite the potential benefits of radiographers’ research engagement, they frequently encounter obstacles that impede their active participation and require adequate support to face and overcome barriers that hinder active involvement. These barriers may include time constraints, limited resources, administrative complexities, and a lack of formal research training. Recognising and addressing these challenges is essential for creating an environment conducive to research engagement [2,3,7,11,12].

Radiographers in the Arab region acknowledge the significance of research in advancing and enhancing their field. Most educational institutions in the Arab world advocate research as a pivotal learning approach to fostering and bolstering practice-based growth. There is a notable scarcity of radiographers pursuing advanced education, resulting in comparatively low research output compared to other professions [16,17,18].

This study seeks to comprehensively understand radiographers’ engagement with practice-based research in Arab countries. Specifically, it investigates the motivating factors driving radiographers’ participation and the barriers hindering their involvement.

## 2. Methodology

**Figure 1:**
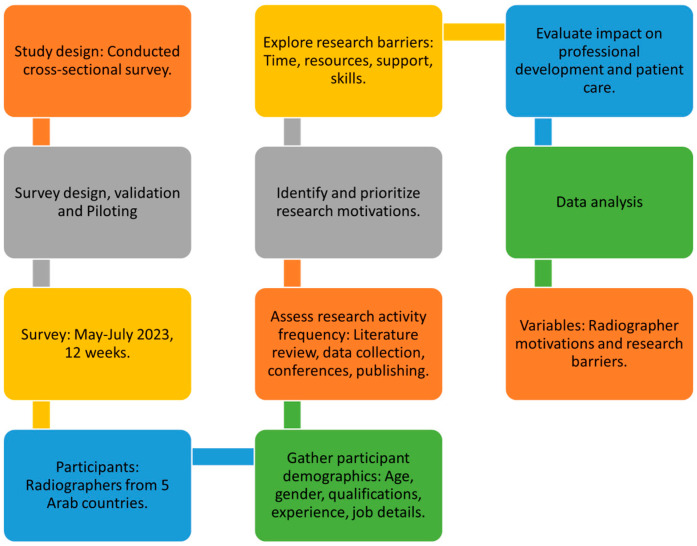
Methodology Flowchart.

This study explores radiographers’ motivations and barriers in their engagement with practice-based research, focusing on five Arab countries. By exploring these factors, the study seeks to understand what motivates radiographers to engage in research and find ways to encourage a culture of research excellence in radiography. The study’s workflow is shown in Figure 1.

### 2.1. Study Design and Data Collection

A cross-sectional study using an anonymous survey using the Google Form platform was developed and distributed online between 1 May 2023 and 31 July 2023 across radiographers working in five Arab countries (Iraq, Kingdom of Saudi Arabia (KSA), Palestine, Sudan, and the United Arab Emirates (UAE)). All responses were anonymous, and submissions were stored in an encrypted form and access-controlled by the principal investigator.

### 2.2. Study Sample

A sample of 250 radiographers from across 5 Arab countries through convenience sampling were included in the study. Fifty participants were chosen from each country to achieve a balanced distribution, which ensures representative insights from diverse healthcare contexts within the region. The researchers approach radiographers through professional networks, healthcare institutions, and relevant associations. To participate in the study, radiographers must be practising and willing to participate voluntarily. The survey was distributed through online survey platforms, and the participants were informed about the study’s purpose, procedures, and the voluntary nature of participation. They received clear instructions on how to access and complete the survey. Informed consent was obtained before participants engaged in the survey, emphasising their right to withdraw at any point without repercussions.

### 2.3. Survey Design

The survey was designed to investigate the factors influencing radiographers’ engagement, barriers, and motivation to participate in research within clinical settings across Arab countries. The survey was divided into seven sections, each addressing different aspects of the research topic. The first section gathers demographic information about the participants, including age, gender, qualifications, work experience, and job details. The second section focuses on research engagement, querying participants about the frequency of their involvement in research-related activities, such as literature reviews, data collection, presenting at conferences, and publishing in journals. The participants were asked to rank the motivating factors for engaging in the research. The third section delves into the barriers that prevent participants from engaging in research more frequently, covering issues like time constraints, resource limitations, lack of support, and insufficient research skills. The fourth section explores participants’ perceptions of how engaging in research impacts their professional development and patient care and outcomes. The fifth section assesses participants’ interest in and opportunities for research involvement, evaluating their motivation and whether they have dedicated time for research during clinical practice. The sixth section asked participants to prioritise motivation factors for research engagement. Finally, the seventh section provided an open-ended space for participants to share their insights on strategies to increase radiographers’ research participation and their general motivations for pursuing research.

The survey instrument used in this study was carefully designed to gather relevant data. Before distribution to the target respondents, the survey underwent a rigorous piloting and testing phase to ensure its reliability and validity. This process involved a group of radiographers and faculty members (radiographers and radiologists) who were not part of the main study, and their feedback was used to refine and improve the survey instrument.

### 2.4. Statistical Analysis

The survey responses were compiled in Microsoft Excel (Microsoft, Redmond, WA, USA) and subsequently subjected to analysis using SPSS statistical software version 27 (IBM SPSS Inc., Chicago, IL, USA). The frequency distributions and the corresponding percentages for each response category were computed. The questions collected more than one response from each respondent, generating cumulative frequencies greater than the sample size. The responses to the 4-point Likert scale were scored from 1–4 to perform parametric analysis and calculate the central tendencies to get insight into the responses. The normality test concluded that the data was heterogeneous in variance and skewed distribution; hence, the Kruskal–Wallis test was conducted to compare radiographers’ responses across different countries. A chi-square analysis was conducted to compare the responses across different demographic settings, and a Pearson’s correlation was conducted to assess any significance between opportunities and motivation.

## 3. Results

The survey resulted in 250 responses, 50 (20.0%) from each of the following five countries: Iraq, KSA, Palestine, Sudan and UAE.

Table 1 presents the study findings across regions’ demographic, professional, and educational aspects. A significant majority falls in the 20–29 age range: Iraq (46%), KSA (56%), UAE (54%), Sudan (32%), and Palestine (56.0%). The 30–39 age group follows with variations: Iraq (32%), KSA (14%), UAE (18%), Sudan (36%), and Palestine (26%). Gender balance was maintained with slightly more females. The 0–10 years’ experience group dominates in Iraq (62%), KSA (62%), UAE (68%), Sudan (54.0%), and Palestine (78.0%). Bachelor of Science qualifications are prevalent, while Iraq stands out in diplomas (36.0%). Radiographers dominate, especially in Iraq (86.0%) and Palestine (84.0%). Sonographers, MRI techs, radiation safety officers (RSOs), and faculty vary by region. Public hospitals have a strong representation, and private hospitals show a notable presence with smaller academic affiliations.

### 3.1. Research Engagement

**Figure 2:**
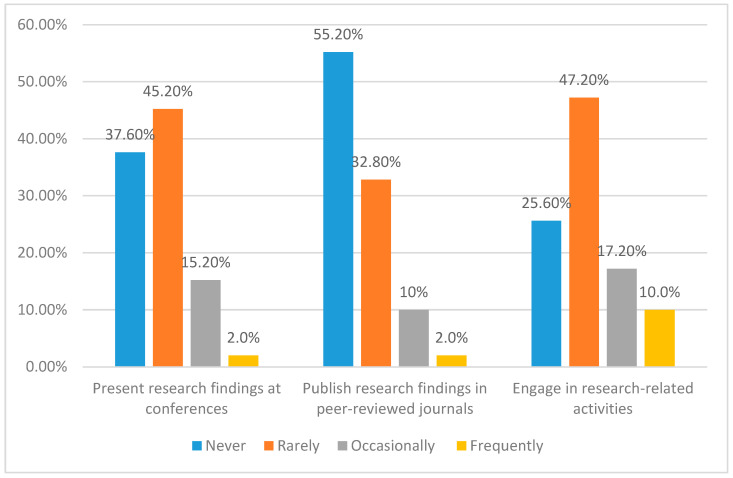
Engagement Levels in Research-Related Activities and Publications.

Figure 2 offers insights into the engagement levels of individuals in research-related activities, including presenting research findings at conferences and publishing research findings in peer-reviewed journals. The data are categorised based on the frequency of engagement, ranging from “Never” to “Frequently (more than four times a year).”

Overall, Figure 2 underscores the participants’ varying engagement in research-related activities. While the “Rarely” and “Never” categories are prominent in presenting at conferences and engaging in research activities, a larger proportion expresses non-involvement in publishing research findings in peer-reviewed journals.

A chi-square analysis was conducted to study the research engagement and contribution of the participants country-wise. There is a significant relationship between the countries and their engagement in research (χ^2^(12) = 58.209, *p* < 0.001). Participants from Sudan (22.0%) were more likely to engage in research frequently compared to others (<10.0%), whereas those from Palestine (46.0%) and Iraq (40.0%) have never engaged in research-related activities.

The chi-square analysis showed a significant association between the countries and the frequency of presenting research findings (χ^2^(12) = 36.822, *p* < 0.001). A total of 24% of participants from Sudan stated that they present more than twice a year, while only 4% of participants from Iraq or Palestine presented as frequently as that. There was also a significant association between the country of participants and the frequency at which they publish their research findings (χ^2^(12) = 38.651, *p* < 0.001). Most participants from Palestine (88.0%) have never published their findings in peer-reviewed journals compared to Sudan and KSA, which only have 44.0% and 40.0% of participants who have never published any study. There also was a significant association between the qualifications of participants and their research engagement (χ^2^(9) = 70.182, *p* < 0.001), presenting findings at conferences (χ^2^(9) = 23.429, *p* < 0.005), and publishing in journals (χ^2^(9) = 38.236, *p* < 0.001). Participants with a PhD qualification were more likely to engage in research (60%) occasionally, present research findings at conferences (32%), and publish in peer-reviewed journals (28%).

**Figure 3:**
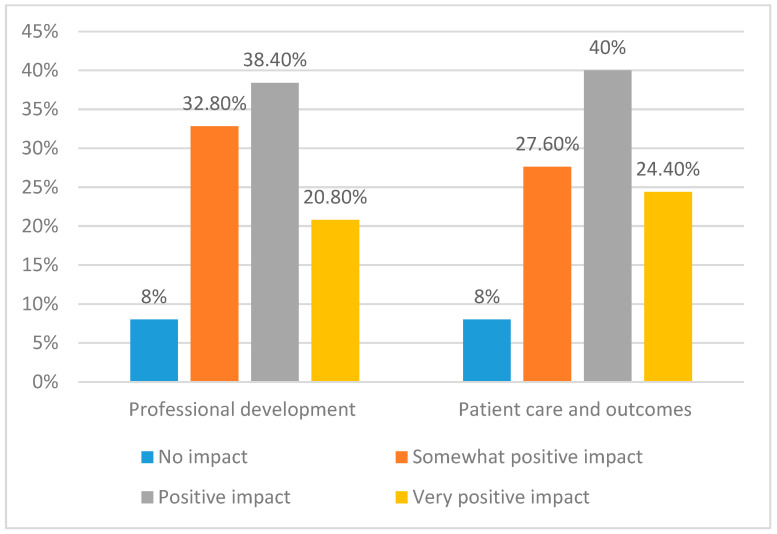
Comparative Analysis of Research Impact.

The impact of engaging in research-related activities in terms of the professional development of the participants and the patient care and outcomes they achieved is represented in Figure 3. The impact was evaluated through categorical responses, “No impact”, “Somewhat positive impact”, positive impact”, and “Very positive impact”. Most of the participants held the view that involvement in research exerts a positive impact on their professional development (34.8%) as well as on patient care and outcomes (40%). Subsequently, 32.8% and 27.6% of the participants posited that research positively impacts their professional development, patient care, and outcomes.

Within the free-text responses on increasing the participation of radiographers in research, many radiographers emphasised that radiographer engagement could be facilitated through financial support, funding, and resources. This sentiment is reflected in statements such as:

“By financial and sources support”, “funding and workshops”, “by providing training in how to do research and offering research activities”, “motivations and training sessions”, and “by providing education.”

Additionally, respondents noted their requirement for dedicated time to engage in research, as evidenced by statements such as:

“By giving them the time from the facilities they work at”, “should have mandatory one or two working days in research”, and “give them the time and resources”.

### 3.2. Research Motivation

Table 2 presents the perceived importance of various factors motivating respondents’ engagement in research. Each factor is rated on a scale from 1 (most important) to 5 (least important). The table includes each factor’s numerical counts, percentages, and statistical measures (mean and standard deviation (SD)).

The factors being assessed are (a): This pertains to the impact of activities on patient well-being. (b) Around 30% of respondents considered it the most important, while a significant portion considered it less important (18% to 20.8%). (c) Recognition and promotion within the profession: this factor reflects the value placed on career advancement and acknowledgement. Around 26.4% of the respondents found it the most important, while 18.8% rated it as the least important. (d) Collaborative working with other healthcare professionals relates to teamwork and cooperation among healthcare practitioners. Approximately 24% of respondents ranked this as the most important, and a similar percentage found it less important. (e) Personal Interest and Curiosity: this factor encompasses intrinsic motivation and curiosity in the field. Around 22.8% of respondents saw it as most important, while 13.6% rated it as least important.

**Figure 4:**
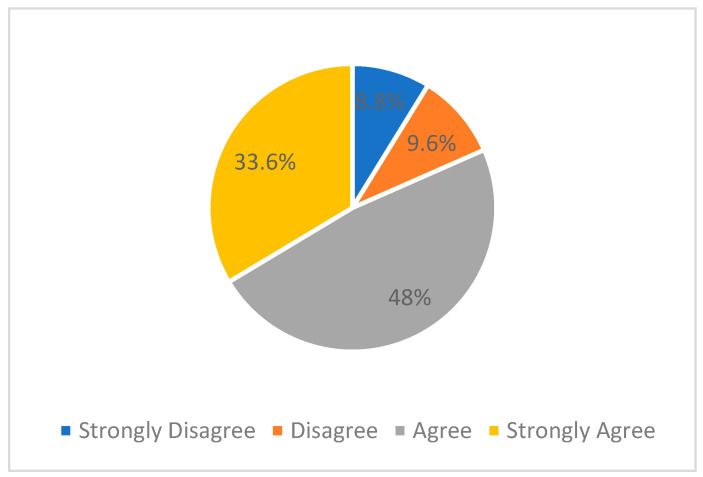
Motivation to work in research.

Furthermore, the participants were asked about their concurrence in being motivated to commence working in research within clinical practice. The responses were gathered on a 4-point Likert scale and scored as follows: Strongly Disagree = 1, Disagree = 2, Agree = 3, and Strongly Agree = 4. Within the cohort of respondents, 81.6% agreed that they were motivated (Figure 4). The Kruskal–Wallis test was employed to analyse the variation in the notion of being motivated across the five countries. The test outcomes revealed a statistically significant difference in the motivation to begin working in research between different countries, chi-square = 13.670, df = 4, *p* = 0.008. The mean motivation scores were Sudan = 148.32, KSA = 135.46, UAE = 122.76, Iraq = 118.74, and Palestine = 102.22.

There was no significant difference in the motivation levels of participants across the age categories (χ^2^(4) = 3.614, *p* = 0.461), gender (χ^2^(1) = 1.214, *p* = 0.271), years of experience (χ^2^(4) = 7.297, *p* = 0.121), job title (χ^2^(4) = 5.818, *p* = 0.213), qualification (χ^2^(3) = 6.584, *p* = 0.086), and their workplace (χ^2^(2) = 2.724, *p* = 0.256).

The subsequent open-ended items captured the participants’ perceptions of the factors that generally motivated them to participate in the research. Among these factors, the predominant motivation sources were financial incentives and opportunities for professional advancement. This inclination is evident in statements such as:

“Possible Increase in Financial Income.” “Acquiring more knowledge and getting better opportunities”, “Money-certificate”, and “practice courses and salary”.

### 3.3. Opportunities and Barriers

**Figure 5:**
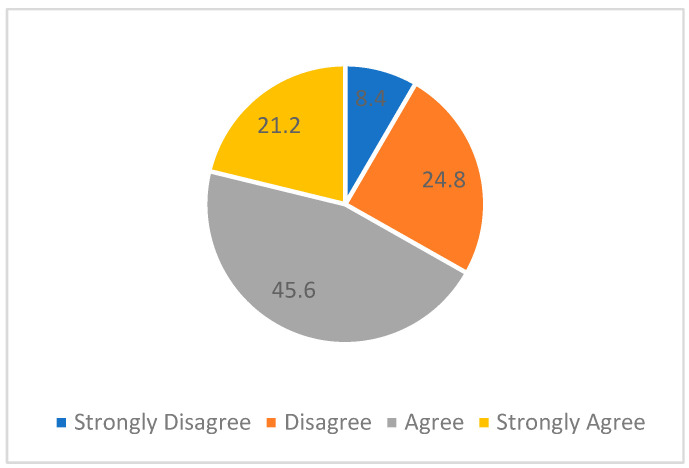
Experienced Opportunities. Data are shown in percentages.

The participants were asked about their alignment with the availability of research opportunities during their clinical practice workday. Responses spanned a 4-point Likert scale from “Strongly agree” to “Strongly disagree” and were assigned scores on a scale from 0–4. Notably, 66.8% of the participants agreed with this proposition, as shown in Figure 5. Moreover, the Kruskal–Wallis test was conducted to ascertain the variation in the availability of opportunities across various countries. The test outcomes revealed a statistically significant difference in the level of agreement concerning the accessible opportunities among the five countries, χ^2^(4) = 20.562, *p* = 0.001. The mean opportunity scores were recorded as follows: Sudan = 160.85, Iraq = 130.33, KSA = 118.60, UAE = 111.83, and Palestine = 105.89. There was a statistically significant difference among the participants who were affirmative on the available opportunities across the age groups (χ^2^(4) = 9.790, *p* = 0.044), gender (χ^2^(1) = 4.183, *p* = 0.041), qualifications (χ^2^(3) = 15.292, *p* = 0.002), and years of experience (χ^2^(1) = 11.628, *p* = 0.020). No statistically significant difference in the perception of opportunities was found across the different job titles (χ^2^(1) = 7.116, *p* = 0.130) and different workplaces (χ^2^(1) = 5.145, *p* = 0.076). The mean rank of those between the ages 50–59 years was the highest at 160.25, and those between 20–29 years was the lowest at 113.05. Male participants had a higher mean rank of 134.71, while the female participants had a mean rank of 117.14. Radiographers with work experience between 0–4 years have a mean rank of 113.47, and those between 21–30 years have a mean rank of 149.46. Participants with a Bachelor’s qualification had a mean rank of 118.99, lower than the rest (135.61–149.06). A statistically significant positive correlation (r = 0.320), *p* < 0.01) was found between motivation and the availability of opportunities, indicating that higher motivation levels were associated with a greater perception of available opportunities.

From a selection of five options, respondents were asked to indicate the barriers impeding their involvement in the research, with the flexibility to choose more than one option. Notably, the participants identified constraints such as insufficient time (56%), restricted resources encompassing aspects like funding and equipment (47.2%), lack of support from peers or superiors (33.2%), lack of knowledge or skills in research methods (32%), and difficulty accessing research literature (18%).

## 4. Discussion

To the best of our knowledge, this study is the first of its kind. It studies the reasons that prevent radiographers from participating in or conducting scientific research in five Arab countries. This is an important step in finding solutions to this problem, where scientific research is a key factor in developing professional identity. The study intends to provide insight into the dynamics that affect radiographers’ involvement in research and pave the way for focused efforts to promote a culture of research-driven excellence within the radiography profession by examining these aspects.

Individuals are involved in research-related tasks such as oral conference presentations and publishing findings in peer-reviewed journals. The results were disappointing, as the highest percentage of participating countries disclosed that they did not or rarely participate in conferences, publish scientific articles, or participate in scientific research. Similar findings from a study by Vikestad et al. in Norway indicated that radiographers are not actively engaged in their research projects. The findings concluded that one of the most important aspects was encouraging radiographers to research to deploy a focused strategy [19]. An illustration of this strategy that Arab countries must take as a role model is the British Radiographer Society’s experience in making crucial decisions to expand scientific research skills by adopting a structured approach to accomplish this objective [20].

One such study examined the reality of the profession’s development in the United Kingdom (UK) and concluded that radiographer involvement in scientific research has significantly increased. In addition, there was a discernible rise in the number of radiographers participating in scientific research, which is a motivating element. Still, there is a need to encourage worthwhile research that improves and supports the work of scientific research in this sector [2]. One researcher considered that traditional radiographers are consumers and non-producers if they need to develop themselves in practical research. They indicated that it is vital to provide aspiring graduates with critical thinking and self-evaluation skills to enable them to succeed in their future healthcare careers [21,22,23,24].

Previous studies found that radiographers holding a PhD degree and working in academia contribute more to research and publication than radiographers holding diplomas and Bachelor’s degrees [1]. Our study results concur with these findings. The simple fact is that only some undergraduate radiography students complete a formal research project during their academic studies. This could lead to radiographers’ lack of involvement in research activities, and this background can influence radiographers’ competence in engaging in research after they finish their education [19]. Radiographers with doctorate degrees may have a higher ability than those with lower degrees to engage in scientific research [4,20,21,25].

Therefore, medical facilities like higher education or hospitals must motivate the development of radiographers’ abilities to perform research and integrate evidence-based information into their clinical practices to alter these viewpoints and innovate services and processes [25,26,27]. The successful execution of the above improvements necessitates multidisciplinary collaboration at the departmental and institutional levels, not simply individual radiographer interests. A study by Knapp et al. found that one of the reasons for radiographers’ inability to engage in scientific research is the need for more investment by higher education institutions in developing research capabilities in this field [24].

A pronounced disparity exists in the levels of engagement and contribution to research activities among the surveyed countries. Notably, radiographers from Sudan exhibit a substantially higher rate of research involvement, motivation, and perceived opportunities to engage in research. In stark contrast, their counterparts in Iraq display considerably lower rates, with only 10% frequently participating in research. Similarly, radiographers from Palestine demonstrate limited engagement in research and comparatively low motivation to engage in the future. Our study revealed that radiographers in Sudan are motivated to engage in research and have the necessary avenues to pursue it. At the same time, those from Palestine lacked the resources and were therefore motivated less compared to other countries. The motivation and research contributions thus far correspond with the extent of research opportunities provided to radiographers, emphasising the need to extend opportunities for clinical radiographers to participate in research. This corresponds with the results of a study in Singapore, where a lack of resources and a heavy workload were identified as the main barriers to research engagement [28].

In this survey, 36.4% of the participants responded that professional development was their primary driving force for conducting research. This result is consistent with a related review by Malamateniou (2009), the author of which concluded that a favourable attitude toward research is necessary for professional growth [2]. Although professional development was reported as a major reason for motivating radiographers to participate in research, it did not show a significant difference compared to other factors, such as personal interest and curiosity, where the latter showed a very close affinity with other factors in terms of values achieved.

Fifty-six percent of respondents in this study found that the main constraint the participants identified in involvement in research was insufficient time. This is consistent with other studies that indicate that a major obstacle to research involvement is a lack of time, indicating radiography’s present weak research profile [7,24,25]. A recent study by Pedersen stated that finding time to start working on significant research tasks, such as developing a research plan, filing authoring articles, and performing research, during ordinary clinical practice may be complex [11]. Other elements considered institutional hurdles were also mentioned in this study, including “limited resources like financing and equipment, lack of support from peers or superiors, lack of understanding or abilities in research methodologies”. This has been confirmed by Reid and Edwards, who revealed that the most important factors that make the research high-quality research are financial support and guidance by the academic supervisor or other radiographer researchers [12].

The study recommends addressing the barriers hindering radiographers’ participation in scientific research in Arab countries, first, by providing structured research training and education, and, second, by developing strategies for effective time management that allow radiographers to balance clinical responsibilities with research activities, encouraging collaboration between radiographers, other healthcare professionals and researchers from various disciplines and countries. By implementing these recommendations, stakeholders in the radiography field can work together to create an environment that fosters a culture of research-driven excellence, ultimately advancing radiographers’ professional identities and capabilities in Arab countries [5,29,30].

## 5. Conclusions

In conclusion, this study, conducted across five Arab countries, identified key motivations and barriers influencing radiographers’ participation in research. Financial incentives and opportunities for professional advancement emerged as primary motivators, while time constraints, resource limitations, lack of support, and inadequate research skills served as significant impediments. Notably, most radiographers desired research opportunities during their practice workday. To foster a culture of research engagement among radiographers, healthcare organisations and policymakers must prioritise support measures such as dedicated research time during clinical practice, training, development opportunities, and financial incentives. Addressing these issues will enhance patient care and outcomes and professional growth.

## 6. Limitation

This study has limitations, including a relatively small sample size and the validation of the instrument, although it was used in a previous study. These limitations will be considered in future research.

## Figures and Tables

**Table 1:** Comparative Analysis of Participant Demographics, Qualification, and Roles.

		Total		Iraq		KSA		Palestine		Sudan		UAE	
		n	%	n	%	n	%	n	%	n	%	n	%
Age	20–29	122	48.8	23	46	28	56	28	56	16	32	27	54
	30–39	63	25.2	16	32	7	14	13	26	18	36	9	18
	40–49	52	20.8	10	20	12	24	8	16	11	22	11	22
	50–59	13	5.2	1	2	3	6	1	2	5	10	3	6
Gender	Male	119	47.6	23	46	18	36	29	58	25	50	24	48
	Female	131	52.4	27	54	32	64	21	42	25	50	26	52
Experience	0–10	162	64.8	31	62	31	62	39	78	27	54	34	68
	11–20	58	23.2	14	28	11	22	8	16	14	28	11	22
	21–30	25	10	4	8	7	14	3	6	8	16	3	6
	31–40	5	2	1	2	1	2	0	0	1	2	2	4
Qualifications	Diploma	18	7.2	18	36	0	0	0	0	0	0	0	0
	B.Sc.	180	72	32	64	42	84	44	88	27	54	35	70
	M.Sc.	27	10.8	0	0	0	0	3	6	11	22	13	26
	Ph.D.	25	10	0	0	8	16	3	6	12	24	2	4
Job title	Radiographer	158	63.2	43	86	24	48	42	84	32	64	17	34
	Sonographer	34	13.6	5	10	11	22	2	4	6	12	10	20
	MRI Tech	28	11.2	2	4	11	22	0	0	2	4	13	26
	RSO	16	6.4	0	0	2	4	2	4	4	8	8	16
	Faculty	14	5.6	0	0	2	4	4	8	6	12	2	4
Public or private hospital	Public	174	69.6	33	66	41	82	32	64	31	62	37	74
	Private	62	24.8	17	34	7	14	14	28	13	26	11	22
	Academic	14	5.6	0	0	2	4	4	8	6	12	2	4

The abbreviations are: KSA—Kingdom of Saudi Arabia, UAE—United Arab Emirates, MRI Tech—Magnetic Resonance Imaging Technologist, and RSO—Radiation Safety Officer.

**Table 2:** Perceived Importance of Factors Influencing Professional Engagement.

	Most Important		Least Important				
	1	2	3	4	5		
	N (%)	N (%)	N (%)	N (%)	N (%)	Mean	SD
Professional development (e.g., advancing knowledge, acquiring new skills)	91 (36.4)	40 (16)	50 (20)	22 (8.8)	47 (18.8)	2.58	1.512
Contributing to patient care and outcomes	75 (30)	47 (18.8)	52 (20.8)	31 (12.4)	45 (18)	2.7	1.466
Recognition and promotion within the profession	66 (26.4)	47 (18.8)	53 (21.2)	40 (16)	44 (17.6)	2.8	1.44
Collaborative working with other healthcare professionals	60 (24)	60 (24)	50 (20)	43 (17.2)	37 (14.8)	2.75	1.381
Personal interest and curiosity	57 (22.8)	48 (19.2)	60 (24)	34 (13.6)	51 (20.4)	2.9	1.433

## Data Availability

The data presented in this study are available on request from the corresponding author.
